# Risk factors leading to the formation of intranasal synechiae (adhesions) in patients who underwent septo- and septoturbinoplasty (a retrospective cohort Study)

**DOI:** 10.1097/MS9.0000000000003223

**Published:** 2025-05-12

**Authors:** Mateusz J. Stępiński, Jacek Banaszewski

**Affiliations:** a Department of Laryngology with Maxillofacial Surgery Subdepartment, Multidisciplinary Regional Hospital, Gorzow Wielkopolski, Poland; b Department of Otolaryngology and Laryngological Oncology, Poznan University of Medical Sciences, Poznan, Poland

**Keywords:** nasal septum, nasal surgical procedures, turbinates

## Abstract

**Introduction:**

Intranasal adhesions (synechiae) are common complications of nasal surgery. This study aimed to analyze the factors influencing the development of synechiae, with particular emphasis on the use of nasal septal splints. We also propose a solution to reduce the incidence of this complication in the future.

**Methodology:**

This retrospective analysis of patients (*n* = 243) who underwent septoplasty and septoconchoplasty between 2017 and 2022.

**Results:**

Iatrogenic intranasal synechiae occurred in 26.75% (65/243) of patients. Among patients who received nasal septal splints, synechiae occurred in 13.1%. Synechiae complicated 46.9% of the surgeries in the group without separators (*P* < 0.001). There was no statistically significant relationship between increased risk of synechiae and concomitant conchoplasty (26% vs. 27.7%, *P* = 0.817). Patients with seton gauze dressings experienced synechiae significantly more often than those with nasal tampons or other gauze dressings (*P* < 0.001).

**Conclusions:**

The use of nasal septal separators and tampons significantly reduces the risk of iatrogenic intranasal synechiae following septoplasty or septoconchoplasty, regardless of the degree of nasal septum deviation. Conversely, the use of seton gauze dressings is associated with a higher risk of developing synechiae.

## Introduction

The nasal septum is the structure that separates the nasal cavity into two parts, right and left. It is composed of three main sections: the membranous part, located between the anterior nares (nostrils); the cartilaginous part, formed by the septal nasal cartilage (also called the quadrangular cartilage); and the bony part, which includes the maxillary bones (forming the crest), the perpendicular plate of the ethmoid bone, and the vomer, along with the vomeroethmoidal suture, a common site for deviations of the nasal septum. These deviations often take the form of a spike^[^1,2^]^. In the anterior-inferior part of the vomer is the vomeronasal cartilage, where Jacobson’s organ (also called the vomeronasal organ, vestigial in humans) is located^[^1^]^. A deviation in the cartilaginous part can have more serious clinical implications, particularly in cases where it significantly reduces nasal cavity patency^[^3^]^.

**HIGHLIGHTS**

- The use of nasal splints and modern nasal dressings reduces the risk of iatrogenic synechiae.
- The use of seton gauze contributes to an increased risk of iatrogenic intranasal adhesions.
- Simultaneous submucosal conchoplasty (turbinoplasty) performed by radiocoagulation (during septoplasty) does not increase the risk of iatrogenic intranasal adhesions.

According to Cottle, the nasal septum is divided into five areas: (1) the nostril, (2) the nasal valve, (3) the area beneath the bony and cartilaginous vault (referred to as the attic), (4) the anterior part of the nasal cavity, which includes the heads of the turbinates and the infundibulum, and (5) the posterior tract of the nasal cavity, including the tails of the turbinates. This system is often used to describe the location of deviations^[^6^]^.

The most important function of the nasal septum, particularly its caudal part, is to provide support for the external part of the nose. Septoplasty should not be performed if the support function is compromised^[^3^]^.

The nasal turbinates (conchae) divide the nasal cavity into three ducts (inferior, middle, upper, and superior in some patients). The inferior nasal concha is an even bone, lying in part of the inferior lateral wall of the nasal cavity, covered with a mucous membrane (rich in blood vessels and mucous glands)^[^2^]^.

## Aim

Analyze the factors influencing the development of intranasal synechiae (adhesions) and suggest solutions to reduce this complication in the future.

## Methods

This retrospective cohort study consisted of a retrospective analysis of the medical records of patients undergoing treatment at the Provincial Laryngology Clinic in XXX (WPL), and at the Department of Laryngology with a Subdivision of Maxillofacial Surgery of the Multispecialist Regional Hospital in XXX (OL) from 2017 to 2022. This project was not a medical experiment; it received institutional ethical approval (number KB – 29/24) and was registered in a public database (NCT06482866). It has been prepared in accordance with the STROCSS criteria^[^7^]^.

**Figure 1.**
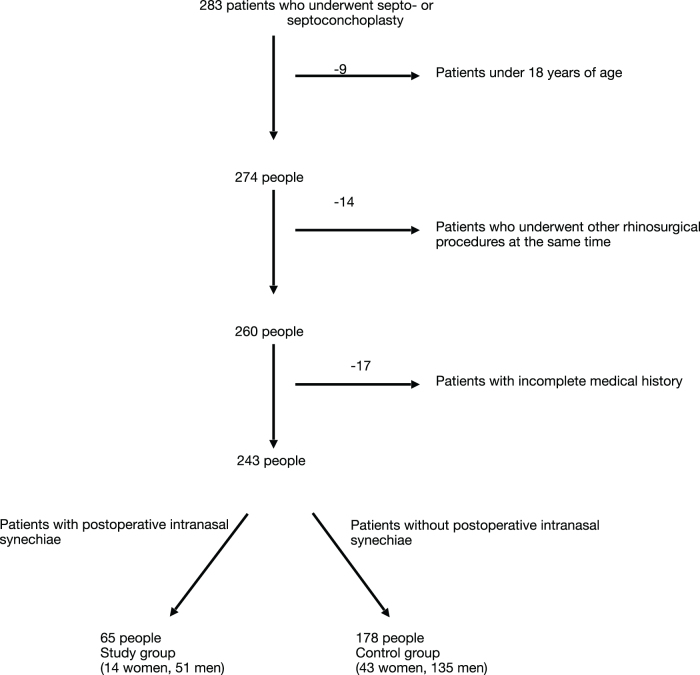
Details of the selection process.

Originally, the study included 283 patients who underwent rhinosurgery: septoplasty and septoconchoplasty (ICD-9 code 21.841) in OL between 2017 and 2022. Nasal septal surgery was performed using Cottle’s method, and conchoplasty was always performed with radiocoagulation using a radiosurgical tool (RaVoR). The authors excluded patients under the age of 18, those with incomplete data, patients operated on in a different department, and those undergoing additional rhinosurgical procedures (except conchoplasty). The authors then closely analyzed the patients’ documentation and divided the population into two groups – control and study. The differentiating criterion was the presence of iatrogenic intranasal synechiae in the patients’ documentation. Finally, 243 people were included in the examination: 65 in the study group and 178 in the control group. Details of the selection process are presented in Figure 1.

All patients were subjected to a preoperative physical examination and medical history was taken, including (on the 3-grade Levine and May scale) the assessment of the degree of nasal septal deviation (NSD) and the presence of inferior HC. Computed tomography (CT) of the paranasal sinuses was not performed.

Detailed characteristics of the study and control groups are shown in Tables 1 and 2, respectively.

**Table 1:** Characteristics of the study group

Patient no.	Sex (F – female, M – male)	Age (on the day of the surgery)	Type of nasal dressing used	Use of nasal septum separators (0 – no; 1 – yes)	Simultaneous conchoplasty (0 – no; 1 – yes)	Preoperative degree of curvature of the nasal septum
1	M	69	TN	0	0	1
2	M	70	GS	0	0	1
3	M	67	GS	0	0	1
4	M	39	GS	0	0	2
5	M	61	GS	0	0	2
6	M	51	GS	0	0	2
7	M	43	GS	0	0	2
8	M	54	GS	0	0	2
9	M	34	GS	0	0	1
10	M	28	GS	0	1	2
11	K	35	GS	0	1	2
12	M	18	GS	0	1	2
13	M	30	GS	0	0	2
14	M	27	TN	0	0	3
15	M	29	GS	0	0	1
16	K	33	TN	0	0	1
17	M	54	GS	0	1	2
18	M	29	GS	0	0	2
19	M	27	TN	1	1	2
20	M	47	TN	0	1	3
21	M	51	GS	0	1	1
22	M	42	TN	1	1	3
23	M	33	GS	0	1	2
24	M	30	TN	1	0	3
25	K	44	GS	0	0	2
26	M	67	GS	0	0	2
27	M	40	GS	0	0	2
28	M	36	GS	0	1	2
29	M	20	GS	0	0	2
30	M	31	GS	0	1	1
31	M	59	TN	1	1	3
32	M	49	GS	1	1	3
33	K	20	GS	0	1	2
34	K	18	TN	1	1	3
35	K	39	TN	1	1	2
36	K	19	GS	0	1	1
37	K	24	GS	0	0	1
38	M	22	GS	0	0	1
39	M	36	GS	0	0	1
40	M	35	GO	1	1	2
41	M	57	TN	1	1	2
42	M	56	TN	0	1	3
43	M	27	TN	0	0	2
44	M	45	GS	0	1	1
45	M	69	GS	0	1	2
46	K	33	TN	1	1	3
47	M	19	TN	1	1	3
48	M	32	GS	0	0	1
49	M	18	TN	1	1	2
50	K	52	GS	1	1	3
51	M	57	TN	1	1	3
52	K	59	GS	0	0	2
53	K	28	GS	0	0	1
54	M	52	TN	1	1	3
55	M	23	GS	0	1	1
56	K	24	GS	0	0	1
57	M	27	TN	0	1	3
58	M	52	GO	1	1	2
59	M	65	GS	0	0	2
60	M	38	GS	0	0	3
61	M	54	TN	1	1	3
62	K	75	GS	1	0	2
63	M	66	TN	1	0	3
64	M	42	GS	0	1	2
65	M	47	GS	0	1	2

**Table 2:** Characteristics of the control group

Patient no.	Sex (F – female, M – male)	Age (on the day of the surgery)	Type of nasal dressing used	Use of nasal septum separators (0 – no; 1 – yes)	Simultaneous conchoplasty (0 – no; 1 – yes)	Preoperative degree of curvature of the nasal septum
1	M	44	GS	1	1	2
2	M	36	GO	0	0	3
3	M	67	GS	0	0	1
4	M	33	GO	1	1	2
5	M	32	GS	0	0	1
6	M	33	GS	0	1	1
7	K	47	TN	0	0	1
8	M	44	GO	1	1	2
9	K	56	GS	0	0	2
10	M	43	GO	1	0	1
11	M	50	GO	1	1	2
12	M	37	GS	0	1	3
13	M	36	TN	0	1	1
14	K	34	TN	1	0	1
15	M	18	TN	1	0	3
16	M	71	GS	0	0	1
17	K	52	TN	1	1	2
18	K	47	TN	0	0	2
19	M	50	TN	1	0	3
20	M	37	TN	1	1	2
21	M	28	TN	0	1	1
22	K	25	TN	1	1	3
23	M	30	TN	1	1	2
24	K	59	GO	1	1	2
25	M	46	TN	1	0	2
26	M	46	GS	0	0	1
27	K	46	TN	1	1	3
28	K	44	GS	0	0	1
29	K	56	GO	1	0	3
30	M	43	GO	1	1	3
31	K	27	GO	1	1	3
32	M	53	TN	1	1	3
33	K	20	GS	1	1	3
34	M	31	TN	1	0	3
35	M	47	GO	1	0	2
36	M	42	GO	1	0	3
37	M	42	TN	1	0	3
38	K	50	TN	1	1	3
39	M	53	GS	0	0	1
40	M	36	TN	1	0	1
41	M	22	TN	1	1	3
42	M	60	TN	1	0	3
43	K	64	GS	0	0	2
44	M	32	TN	1	0	2
45	M	61	GO	1	1	2
46	M	56	TN	1	1	3
47	M	28	TN	1	1	3
48	M	34	TN	1	1	3
49	M	21	TN	1	1	3
50	M	46	GS	0	1	2
51	M	44	TN	1	0	2
52	M	18	TN	1	0	3
53	K	64	GS	0	0	2
54	K	47	TN	1	1	3
55	M	62	GS	0	0	1
56	M	40	TN	1	0	2
57	M	39	TN	1	0	2
58	M	52	TN	1	0	2
59	M	38	TN	1	0	2
60	M	58	TN	1	0	2
61	M	64	TN	1	1	2
62	M	29	TN	1	1	2
63	M	48	TN	1	1	2
64	K	23	GS	0	1	1
65	M	18	GS	0	1	1
66	M	32	TN	1	1	3
67	M	64	TN	1	1	3
68	M	56	TN	1	1	3
69	M	76	TN	1	0	3
70	M	32	GS	0	1	2
71	M	27	TN	1	1	3
72	K	29	TN	1	1	3
73	M	67	TN	1	1	2
74	M	18	TN	1	0	2
75	M	31	GO	1	1	3
76	M	36	GS	0	0	2
77	M	31	GS	0	0	2
78	M	49	GO	1	1	3
79	M	29	TN	1	1	3
80	M	55	TN	1	1	3
81	K	42	TN	1	1	3
82	M	21	Brak	1	0	2
83	M	35	TN	1	0	2
84	M	28	TN	1	0	2
85	M	37	GO	0	0	2
86	M	71	TN	1	0	2
87	M	69	TN	1	1	3
88	M	23	TN	1	1	2
89	M	27	TN	1	1	3
90	M	34	TN	1	0	3
91	M	29	TN	1	1	3
92	K	68	GS	0	1	1
93	K	33	TN	1	0	2
94	M	37	TN	1	1	3
95	M	36	GS	0	0	1
96	M	34	TN	1	0	2
97	K	52	GS	0	1	2
98	M	62	GO	0	0	3
99	M	36	GO	1	0	3
100	M	40	TN	1	1	3
101	M	40	GS	0	0	2
102	M	31	TN	1	1	3
103	K	40	TN	1	0	2
104	K	70	TN	0	0	2
105	M	38	GS	0	0	1
106	M	47	GS	0	0	1
107	K	44	TN	1	1	3
108	M	54	GS	0	0	3
109	M	36	TN	1	1	3
110	M	32	TN	1	1	3
111	K	27	TN	1	0	2
112	M	39	TN	1	1	2
113	K	50	TN	1	0	2
114	K	45	TN	1	1	3
115	M	55	TN	1	1	2
116	M	29	TN	1	1	2
117	M	36	TN	1	0	2
118	M	22	TN	1	0	2
119	M	60	GS	0	0	1
120	M	23	GS	0	0	3
121	M	19	TN	1	1	3
122	M	33	TN	1	0	2
123	M	51	GS	0	0	2
124	K	34	GO	0	1	3
125	M	62	TN	1	1	3
126	K	52	GS	0	0	2
127	K	20	GS	0	0	2
128	M	43	TN	1	1	2
129	M	37	GS	0	1	3
130	M	27	GS	0	0	3
131	M	28	TN	1	0	2
132	M	61	GO	1	1	2
133	K	32	GO	1	1	2
134	K	19	GS	1	1	2
135	M	25	TN	1	1	3
136	M	61	TN	1	1	2
137	K	45	TN	1	1	3
138	M	44	TN	1	1	3
139	K	38	GS	0	0	2
140	M	53	TN	0	1	3
141	M	55	GO	1	1	2
142	M	51	GO	1	1	3
143	M	53	TN	1	0	3
144	M	33	GS	0	0	1
145	M	22	TN	1	0	2
146	M	63	TN	1	1	3
147	M	41	TN	1	1	2
148	M	56	TN	0	0	2
149	M	45	TN	1	1	2
150	M	42	TN	1	1	3
151	K	32	TN	1	1	3
152	K	29	TN	1	1	2
153	M	45	GO	1	1	3
154	K	39	TN	1	1	2
155	K	30	GO	1	1	2
156	M	32	GO	1	1	3
157	M	67	TN	0	0	2
158	M	29	GS	0	0	2
159	M	62	GO	1	1	3
160	M	33	TN	1	1	3
161	K	51	GS	0	1	2
162	M	65	GO	1	1	3
163	M	31	GS	0	0	2
164	K	33	GS	0	1	2
165	M	37	GO	1	1	2
166	M	27	GO	1	0	3
167	M	69	TN	1	0	2
168	M	29	TN	1	0	2
169	K	46	GS	0	0	2
170	M	62	GS	0	0	2
171	M	60	TN	1	0	3
172	M	47	TN	1	0	2
173	M	30	TN	1	1	2
174	M	39	TN	1	1	2
175	M	62	TN	1	1	2
176	M	24	TN	1	1	2
177	K	55	TN	1	1	3
178	M	51	TN	1	1	2

### Description of the surgical method

The surgical field was washed with a disinfectant (skin of the nose, cheeks, and lips, Skinsept, and nasal cavities, Skinsept Mucosa) under general anesthesia. In the first stage, after local anesthesia of the inferior nasal turbinates and (bilaterally) the nasal septum mucosa with 1% lignocaine solution, a Cottle’s incision was made on the side of the deviation of the nasal septum. Subperichondrial and subperiosteal tunnels were then made, releasing cartilage and bone tissue and reaching the site of the deviation. After mobilization, the excess cartilage and bone were resected and, if necessary, after preparation with a crush (Karl Storz 523900 Cottle Bone Crusher), the cartilage was replanted and fixed with sutures (depending on the situation: to the anterior spike, the mucosa [septal suture] or the remaining fragments of the cartilaginous part), and the mucosa was sutured (PGLA LACTIC, multifilament, 3/0 USP absorbable sutures; 1/2 circle needle, 22 mm).

In selected patients, the nasal splints were installed and conchoplasty was performed.

Nasal splints made of fluoroplastic (Spiggle & Theis, Septum splint, FEP; 0.25–0.75 mm) were used as nasal septal separators (installed on the right and left sides of the nasal septum). The splints were fixed using preseptal sutures.

Conchoplasty was performed by radiocoagulation using an inner electrode. Punctures were made in the direction from the head to the tail of the inferior nasal turbinate, every approximately 15 mm, activating the device for a maximum of 7 seconds.

In the final stage, the nasal cavity was secured using an anterior tamponade. The choice of dressing is at the discretion of the operator. Intranasal dressings were used as follows:

- nasal tampon (TN; Spiggle & Theis, Expanding nasal tampon PVA, straight, sterile, 80 × 15 × 20 mm or 45 × 15 × 20 mm)
- dressing gauze soaked in a hydrocarbon gel (GO; Hartmann, Tampograss, 2 cm × 5 m)
- seton gauze (GS) (Matopat, Seton, 1 cm × 2 m).

After surgery, the patients were transferred to the recovery room where they were transferred to the OL. The nasal dressings were removed on the second postoperative day. On the same day, the patient was discharged for outpatient follow-up.

During the first follow-up visit, on the seventh postoperative day, the nasal splints were removed.

In the postoperative period, all patients were advised to use irrigation of the nasal cavities with isotonic sodium chloride solution (3–5 times a day) and to moisturize the nasal cavities with over-the-counter preparations to be chosen according to individual patient preference (at least 2 times a day).

Outpatient follow-up was performed from the 1st week to the 12th month after surgery, during which attention was paid to the formation of clinically significant (affecting patients’ dissatisfaction with nasal cavity patency) synechiae between the nasal septum and inferior nasal turbinate.

Between 2017 and 2022, seven otorhinolaryngologists, including two residents, performed surgery on OL.

### Statistical analyses

Statistical analyses were conducted using IBM SPSS software. In this study, the authors employed basic descriptive statistics for quantitative data, while qualitative information was presented as a percentage distribution. The level of statistical significance was set at *P* < 0.05.

To assess the relationships between nominal variables, contingency tables were created, and chi-square tests were applied.

The area under the curve (AUC) was used to assess the discriminatory power of test. AUC values range from 0.5 (no discrimination) to 1 (perfect discrimination). Higher AUC values indicate better diagnostic or predictive performance.

## Results

### General characteristics of the population

The study group consisted of 65 patients, including 14 women and 51 men, aged 18–75 years (mean: 41 years; median: 39 years). The control group included 178 subjects, including 43 women and 135 men, aged 18–76 years (mean: 42 years; median: 40 years).

**Figure 2.**
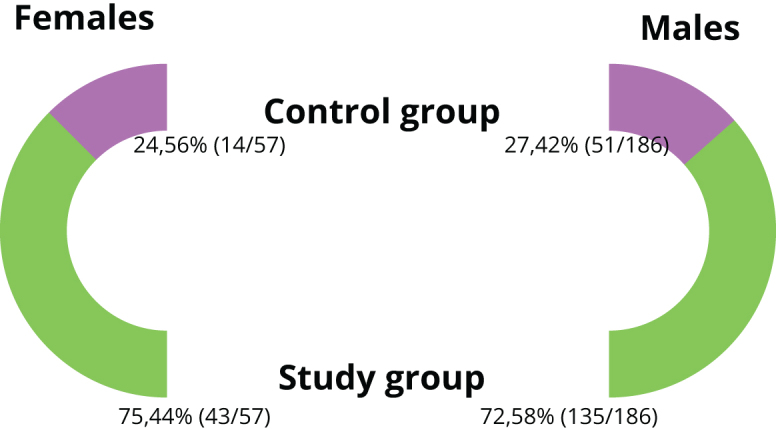
Division of the study and control groups by gender.

Complications in the form of iatrogenic intranasal synechiae occurred in 26.75% (65/243) of patients (K, 24.56% [14/57]; M, 27.42% [51/186]) undergoing septoconchoplasty. There was no statistical relationship between sex and risk of synechiae (*P* = 0.670). The data are presented in Figure 2.

**Figure 3.**
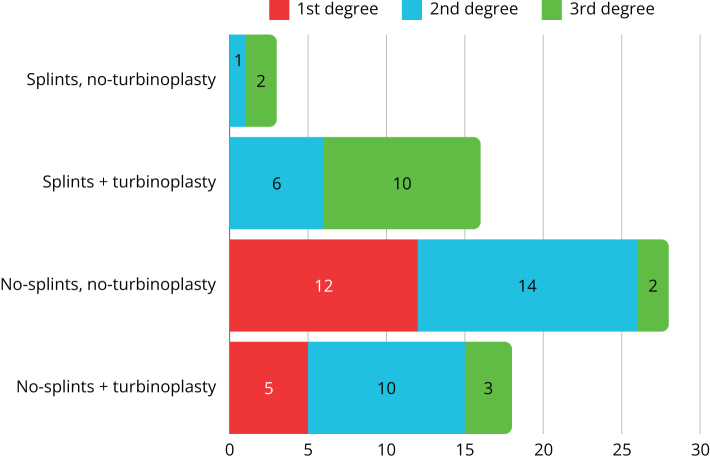
Characteristics of the study group due to the use of nasal septal separators, simultaneous turbinoplasty and the degree of (preoperative) nasal septal deviation.

**Figure 4.**
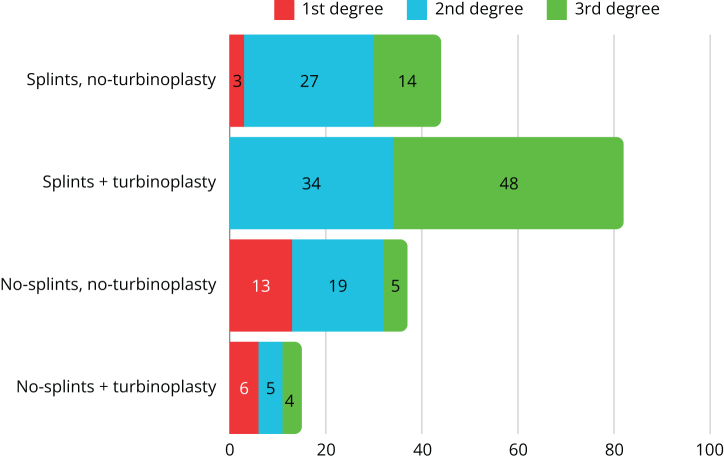
Characteristics of the control group due to the use of nasal septal separators, simultaneous turbinoplasty and the degree of (preoperative) nasal septal deviation.

Figures 3 and 4 show the analysis of the two groups in the context of: the degree of preoperative curvature of the nasal septum, the modification of the septoplasty procedure involving simultaneous conchoplasty, use of the splints.

The study group was predominantly composed of patients with first and second degrees of deviation (26.15%, 17/65, and 47.69%, 31/65, respectively), while third-degree deviation was demonstrated in 26.15% (17/65) of patients. The control cohort was characterized by a different proportion, where patients with grade 1 deviation accounted for 12.35% (22/178), while grades 2 and 3 deviation were described in 47.76% (85/178) and 39.89% (71/178) of eligible patients, respectively.

Nasal splints were used in 19 out of 65 subjects (29.23%; 7/65 with second-degree and 12/65 with third-degree deviation) in the study population, and in 126 out of 178 subjects (70.79%; 3/126 with first-degree, 61/126 with second-degree, and 62/126 with third-degree deviation) in the control group.

Conchoplasty was performed in 52% (34/65) of the study cohort and in 54% (97/178) of the control population.

TN was the most commonly used dressing in the control group (60.11%; 107/178), in contrast to the study group, where GS was the most frequently used nasal dressing (64.62%; 42/65).

The study showed a tendency among operators to use nasal septal separators in cases of grade 3 NSD (34% [62/178] of the control population vs. 18% [18/65] of the study cohort). An inverse correlation was observed when the deviation was in degrees 1 and 2 (63%, 41/65 of the study population vs. 24%, 43/178 of the control group).

The two populations were divided according to the following variables: (1) use of nasal splints, (2) concomitant conchoplasty, (3) type of nasal dressing, (4) degree of nasal septum curvature, and (5) the surgeon who performed the operation. The authors also analyzed the structure of comorbidities.

### Use of nasal septal splints and risk of intranasal synechiae

Among patients in both the study and control groups who received splints, synechiae occurred in 13.1% of cases. In contrast, in the group of patients who did not undergo the modification, synechiae complicated 46.9% of surgeries. A statistically significant relationship between these variables was observed (AUC = 0.669, *P* < 0.001).

### Simultaneous conchoplasty and the risk of intranasal synechiae

The study found no statistically significant relationship between the increased risk of synechiae and concomitant conchoplasty (AUC = 0.509; *P* = 0.817). Synechiae occurred in 26% of patients who underwent concomitant conchoplasty, compared to 27.7% in those who did not.

### Type of nasal dressing used and risk of intranasal synechiae

This study confirmed a statistically significant relationship between the type of nasal dressing used and the risk of intranasal synechiae. Due to an insufficient sample size, patients with no dressing were excluded from the analysis. Synechiae occurred significantly more often in patients with GS dressings compared to those with TN or GO (*P* < 0.001; Table 3). Furthermore, there was a statistically significant reduction in the risk of intranasal synechiae when separators and TN dressings were used (AUC = 0.723; *P* < 0.001). However, it could not be determined whether this reduction was due to the dressing alone or an overlapping effect of both factors.

**Table 3:** The structure of the used nasal dressing

	TN	GO	GS	Without dressing
Study group (*n* = 65)	32.30% (21/65)	3.08% (2/65)	64.62% (42/65)	0% (0/65)
Control group (*n* = 178)	60.11% (107/178)	15.17% (27/178)	24.15% (43/178)	0.5% (1/178)
*P*-value	*P* < 0.001^a^	*P* < 0.001^a^	*P* < 0.001	
Chi-square test	130.249			

^a^ It could not be determined whether this was an effect of the dressing alone or an overlapping effect of both factors (nasal splints).

### Failure to use nasal splints and the use of dressing gauze (as a nasal dressing), and the risk of intranasal synechiae

Synechiae were present in 49.4% of patients who received GS dressings without a splint, while synechiae occurred in only 15.9% of patients who had a separator but no GS. A statistically significant correlation was found between an increased risk of intranasal synechiae and the absence of a splint combined with the use of GS as a dressing (AUC = 0.332; *P* < 0.001).

### Degree of curvature of the nasal septum and risk of intranasal synechiae

Among patients with third-degree curvature of the nasal septum, postoperative synechiae were the least common (19.3%), whereas those with first-degree curvature had the highest incidence (43.6%). A higher degree of septal curvature did not increase the risk of synechiae (*P* = 0.017).

### Structure of operators and the nasal dressing and nasal splints used by them

Table 4 presents the structure of both the cohort of operators and the types of nasal dressings and nasal splints used by them. The otorhinolaryngologists, labeled as A–E, were experienced surgeons, each with a minimum of 5 years of surgical experience (with surgeons A–C having over 20 years of experience). Doctors labeled as F and G were younger, undergoing specialized training, and operated under the supervision of experienced specialists.

**Table 4:** Structure of operators and the nasal dressing and nasal splints used by them

	Case group (*n* = 65)	Control group (*n* = 178)	Case group – use of nasal splints	Control group – use of nasal splints	Percentage of synechiae
A (*n* = 37)					
TN	2	14	6 (100%)	31 (100%)	16.22% (6/37)
GO	2	1			
GS	2	16			
B (*n* = 71)					
TN	2	0	0 (0%)	0 (0%)	47.89% (34/71)
GO	0	1			
GS	32	36			
C (*n* = 15)					
TN	1	2	0 (0%)	2/8 (25%)	46.67% (7/15)
GO	0	4			
GS	6	2			
D (*n* = 1)					
TN	0	1	0 (0%)	1 (100%)	0%
E (*n* = 74)					
TN	7	53	4/9 (44.44%)	55/65 (83.33%)	12.16% (9/74%)
GO	0	8			
GS	2	3			
Without dressing	0	1			
F (*n* = 41)					
TN	8	32	8 (100%)	33 (100%)	19.51% (8/41)
GO	0	0			
GS	0	1			
G (*n* = 4)					
TN	1	3	1 (100%)	3 (100%)	25% (1/4)
GO	0	0			
GS	0	0			

Intranasal synechiae were identified after surgeries performed by surgeons B and C in 47.89% and 46.67% of their operated patients, respectively. These surgeons most often used GS (as a nasal dressing) and less frequently used nasal splints.

Apart from surgeon D (who operated on only one patient), intranasal synechiae were least frequently identified in patients operated on by surgeon E (12.16%). Surgeon E frequently used nasal splints and TN as a nasal dressing.

### Comparison between specialists and residents

**Figure 5.**
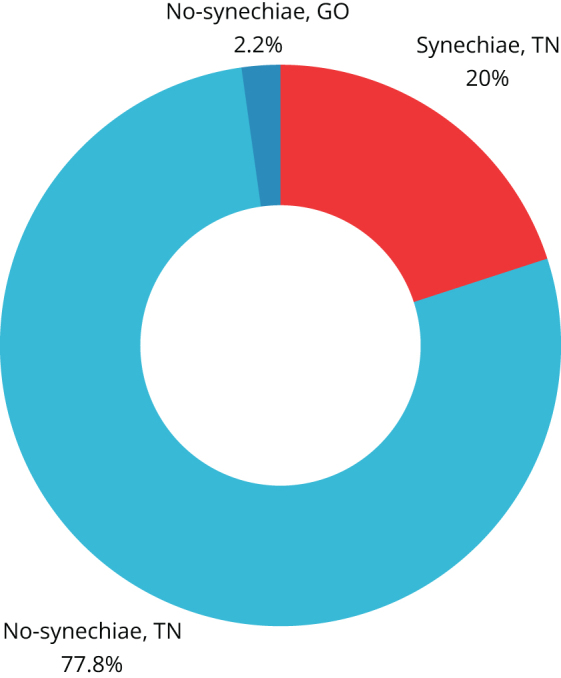
Residents group, structure of synechiae, and type of dressing.

**Figure 6.**
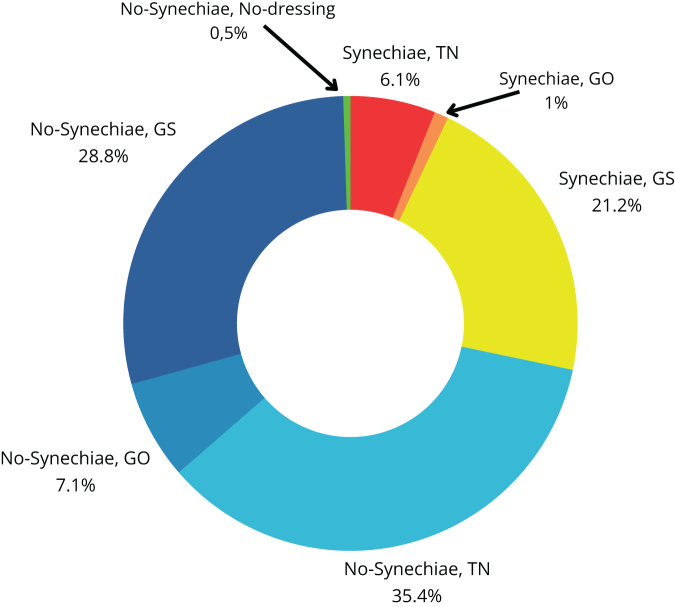
Specialist group, structure of synechiae, and type of dressing.

Table 5 and Figures 5 and 6 compare two groups of operators: residents and specialists. The most commonly used nasal dressing in the specialist group was GS (99/198 cases), with synechiae diagnosed in 28.28% of patients (56/198). Residents, on the other hand, preferred TN, with synechiae identified in 20% of cases (9/45).

**Table 5:** Comparison between specialists and residents: nasal dressings, splints usage, synechiae

	Case group (*n* = 65)	Control group (*n* = 178)	Case group – use of nasal splints	Control group – use of nasal splints	Percentage of synechiae
Specialist (*n* = 198)					
TN	12	70	10/56 (17.86%)	89/142 (62.68%)	28.28% (56/198)
GO	2	14			
GS	42	57			
Without dressing	0	1			
Residents (*n* = 45)					
TN	9	35	9/9 (100%)	36/36 (100%)	20.00% (9/45)
GO	0	0			
GS	0	1			

### Comorbidities

Table 6 presents the structure of comorbidities. In the control group, 61/178 (34.37%) subjects reported comorbidities (27/178 and 15.17%, respectively). The most common comorbidity was hypertension (62.30%, 38/61). Less common comorbidities were obesity, lipid disorders, bronchial asthma, hypothyroidism (9.84%, 6/61), type 2 diabetes, benign prostatic hyperplasia (6.56%, 4/61). Other comorbidities were indicated less often.

**Table 6:** Structure of comorbidities in the study and control groups

Study group (*n* = 17)	Control group (*n* = 61)
Hypertension 52.94% (9/17)	Hypertension 62.30% (38/61)
Obesity, benign prostatic hyperplasia, bronchial asthma (11.76%, 2/17)	Obesity, lipid disorders, bronchial asthma, hypothyroidism (9.84%, 6/61)
Others 5.88% (1/17)	Type 2 diabetes, benign prostatic hyperplasia (6.56%, 4/61)
	Others 1.64% (1/61)

In the study group, 17/65 (26.15%) patients had comorbidities (6/65 patients, 9.23% reported multimorbidity). The most common comorbidity was hypertension (52.94%, 9/17). Less common comorbidities were obesity, benign prostatic hyperplasia, and bronchial asthma (11.76%, 2/17). Other comorbidities were indicated less often

In both cohorts, the most common comorbidity was hypertension.

## Discussion

Deviation of the nasal septum (NDS) and hypertrophy of the nasal conchae (HC) are classified under ICD-10 codes J34.2 and J34.3, respectively. Up to 80% of the population does not have a medially aligned nasal septum^[^5^]^, and approximately 20% of individuals have a deviation that is clinically significant, with about one-quarter of patients reporting clinical symptoms^[^8^]^.

According to Levine and May, NSD is classified on a three-stage scale as follows: (1) the lateral and medial surfaces of the middle nasal concha were visible; (2) the middle nasal concha was partially obscured by a crooked nasal septum; (3) a deformed/curved nasal septum completely obscures the middle nasal concha^[^9^]^. It should be noted that there are various classifications of NSD. For example, Mladina proposed a seven-type classification based on the location of the deviation and its relation to the lateral wall of the nasal cavity^[^10^]^.

**Figure 7.**
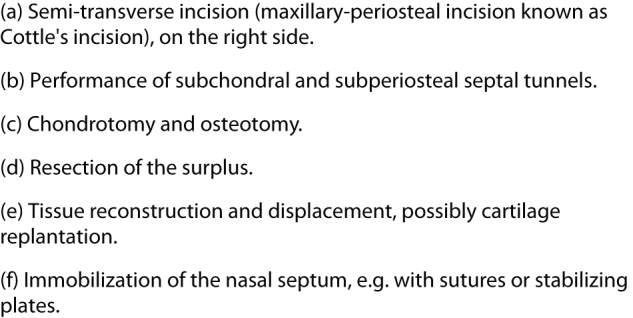
Stages of Cottle’s septoplasty.

The literature describes several methods of nasal septal surgery; endoscopic methods (using 0°, 30°, and 70° rigid optics) and conventional, classical methods are distinguished^[^11^]^. Among the classical methods, the literature draws a clear line between submucosal nasal septal resection (according to Killian) and septoplasty, as described by Cottle – the stages of the operation are described in detail in Figure 7^[^3^]^.

Hypertrophy and deformities in the inferior nasal turbinate, especially symptomatic, are indications for surgical treatment. The literature distinguishes several surgical methods within the inferior nasal turbinates. During a simple turbinectomy, part of the concha’s mucosa is removed, sometimes along with a portion of the underlying bone. An alternative procedure is turbinoplasty (conchoplasty), where the mucosal cover should not be injured, only the deeper layers of the mucosa. This technique employs tools such as lasers, electrocautery, or radiofrequency coagulation. Turbinoplasty is especially helpful during operations on the heads of the conchae^[^3^]^.

The complications that occur after nasal septum surgery as well as inferior nasal conchoplasty include bleeding from the surgical wound, excessive drying of the mucosa, perforation of the nasal septum, and intranasal adhesions (so-called synechiae) between the nasal turbinates and the lateral and medial walls of the nasal cavity – according to the literature, this complication affects 4%–36% of patients^[^4,12,13^]^.

Given the prevalence of the condition and the number of patients undergoing septoconchoplasty, we attempted to analyze the factors influencing the development of intranasal adhesions (synechiae), with particular emphasis on the use of the splints, and to suggest a solution to reduce this complication in the future^[^14^]^.

Initially, splints (separators) were handmade, with early examples including those crafted from X-ray film (1955, Salinger and Cohen) and polyethylene coffee cup lids (1969, Wright). With advancements in bioengineering, splints began to be produced from materials such as polytetrafluoroethylene (Teflon) and polyethylene (1977, Doyle). Other innovations included magnetic splints (1982, Goode, using samarium cobalt magnets) and dental wax (1955, Nayak). Eventually, absorbable materials were introduced for splints, including subepithelial options^[^15^]^. Today, the most commonly used types of separators are made from fluoroplastic (e.g. Reuter bivalve splints) and silicone (e.g. Doyle splints, which allow for air passage)^[^16^]^.

Synechia (adhesion) refers to the connection between two structures, often formed from fibrin or collagen. The creation of scar tissue during the repair process is a natural response, though it can sometimes impair normal function. Occasionally, synechiae develop in undesirable locations, leading to side effects. Traditionally, synechiae are considered complications following abdominal surgery, but they can also occur in other body cavities, such as the nasal cavity or nasopharynx^[^17–19^]^.

Damage to the deeper layers of tissue, including the mucosa, triggers an inflammatory process, which activates fibroblasts and the coagulation cascade. As a result, fibrin and collagen are produced. Microscopic foreign bodies in the surgical area may play a role in this process^[^17,18,20^]^.

In some cases, synechiae may be broken down by fibrinolytic enzymes. However, due to ongoing inflammation (even sterile inflammation) and the activity of macrophages from damaged blood vessels, the formation of synechiae may continue^[^17,19,20^]^.

A percentage of intranasal synechia was observed at the border, similar to other departments. The literature indicates that 3.42%–36% of patients who underwent rhinosurgical treatment (including septoconchoplasty) experienced this type of complication^[^12,13,21^]^.

The marked difference between both populations in terms of preoperative NSD was most likely due to the following: (1) Operating strategy: Grade 1 deviations were operated less invasively, as compared to grades 2 and 3 deviations, where septal separators and TN were much more readily used. (2) Grade 1 deviations do not always produce clinical symptoms and patients are less likely to qualify for nasal septal surgery.

The marked difference between the cohorts (study and control) in the use of nasal splints (29.23% vs. 70.79%) suggests that the present modification reduces the occurrence of intranasal synechiae following nasal septal surgery. This observation has been noted in the literature: the use of medical-grade nasal splints increases the risk of developing intranasal synechiae^[^14,22^]^.

It should be noted that there is no consensus on the optimal time for removing nasal splints. An animal study (rabbits) demonstrated that splints influence the nasal mucosa by generating an inflammatory process; the most compromising period is between 5 and 10 days post-surgery^[^23^]^.

Removing splints after 15 days significantly increased the risk of complications. A study in humans suggested that earlier removal (2–7 days) may be advantageous compared to 10 days, though these findings were not statistically significant^[^24–26^]^.

The results of the study allow us to conclude that conchoplasty performed simultaneously with radiocoagulation, does not increase the risk of iatrogenic intranasal synechiae; the procedure, by design, is based on surgery in the submucosal layer, with minimal damage to the nasal turbinate mucosa, which is reflected in the reduction of complications. Literature confirms that combined septoconchoplasty, compared to septoplasty alone, is associated with a lower risk of iatrogenic long-term complications (such as synechiae). Additionally, septoconchoplasty results in better nasal patency^[^27–29^]^.

The conclusions of the present study are in line with the observations of other researchers: The use of modern nasal dressings (dressing gauze, nasal swab, etc.), compared to the traditional seton gauze, to a lesser extent, leads to damage to the mucosa, and consequently reduces the risk of complications, including intranasal synechiae^[^30,31^]^. In addition, seton gauze dressings can more easily migrate toward the choana, leading to increased discomfort and, in some cases, dyspnea or even death^[^32^]^. Moreover, an animal study (rabbit) demonstrated that properly applied dressings like Merocel can help preserve the normal function of the nasal mucosa^[^23^]^.

The hypothesis that there is no correlation between the preoperative degree of curvature of the nasal septum and the risk of synechiae is debatable and probably due to individual surgical tactics and additional factors (use of separators, dressings, etc.)^[^27,33^]^. This problem certainly needs to be analyzed more thoroughly.

Results from other studies have shown a correlation between surgeon experience and the therapeutic outcomes of surgery, as well as the occurrence of iatrogenic complications^[^34,35^]^. However, our analysis did not confirm the intuitive hypothesis that younger, less experienced doctors would have worse outcomes. This is likely because younger surgeons employed modern dressings (which are safer for the mucosa) and used splints. Synechiae occurred significantly more often in patients operated on by surgeons who did not use splints and opted for GS nasal dressings.

Several chronic diseases increase the risk of intranasal synechiae, including autoimmune conditions such as granulomatosis with polyangiitis and sarcoidosis, as well as infections like leprosy and tuberculosis^[^36^]^. The most commonly identified comorbidity in this study was hypertension, while other conditions were recognized incidentally. The literature does not provide evidence of a direct correlation between hyperthyroidism or hypertension and an increased risk of intranasal diseases.

Smoking is widely recognized as a harmful factor that increases the risk of various diseases, including cardiovascular conditions and cancers. Additionally, it impairs tissue oxygenation, which is crucial for the wound-healing process, particularly during the postoperative period. Smoking also plays a role in the pathogenesis of rhinosinusitis^[^12,37^]^. However, studies have not shown significant differences in postoperative outcomes or complications, such as synechiae, between smokers and non-smokers^[^38–41^]^. In our study, this aspect was not analyzed due to incomplete data in both cohorts.

The literature presents various strategies to reduce the risk of intranasal synechiae after rhinological surgical procedures that were not examined in this study. The beneficial role of irrigation, particularly during the postoperative period, is well recognized, as it aids in cleaning and removing necrotic tissue, which is beneficial for ciliary function^[^42^]^. Notably, the use of hypertonic seawater is considered the best option. Additionally, in challenging cases, the use of mitomycin C (a cytostatic drug used in cancer therapy that exhibits anti-proliferative effects against fibroblasts) can serve as a preventive measure, especially when the anatomy of the nasal cavity is complex, or in situations where synechiae persist despite conventional treatments^[^5,43^]^.

## Limitations of the Study

1. Although NSD is recognized during clinical examination, the lack of a CT scan of the nasal cavity and sinuses prevented an objective assessment of the nasal septum and inferior nasal turbinate hypertrophy.
2. The performance of procedures by several operators, despite the maintenance of template elements of the procedure, leaves a margin for the subjective factor.
3. The use of different types of nasal dressings may have an impact on the final effect and the comparison of different groups.
4. Lack of an objective, non-invasive tool to verify the implementation of postoperative recommendations (based only on the history collected from patients).
5. The marked disparity in the control group in terms of the degree of deviation of the nasal septum may hinder the formulation of final conclusions about the impact of this parameter on the risk of synechiae.
6. No objective classification of synechiae (adhesions); no objective comparison of the study population is possible.
7. Incomplete data regarding smoking.

## Applications

1. The use of nasal septal separators and tampons reduces the risk of iatrogenic intranasal synechiae (adhesions) after septoplasty or septoconchoplasty, regardless of the degree of deviation of the nasal septum.
2. The use of seton gauze contributes to an increased risk of iatrogenic intranasal synechiae after septoplasty or septoconchoplasty.
3. Simultaneous submucosal conchoplasty performed by radiocoagulation (during septoplasty) does not increase the risk of iatrogenic intranasal synechiae.
4. This work allows us to assume that the degree of deviation of the nasal septum does not affect the formation of postoperative intranasal synechiae after septoconchoplasty.

## Data Availability

Analyzed data during the current study are NOT publicly available.

## Research registration unique identifying number (UIN)

Clinical Trials (ClinicalTrials.gov), Nr NCT06482866, https://clinicaltrials.gov/study/NCT06482866?id=NCT06482866&rank=1.
